# A Novel Methodology for Series Arc Fault Detection by Temporal Domain Visualization and Convolutional Neural Network

**DOI:** 10.3390/s20010162

**Published:** 2019-12-26

**Authors:** Kai Yang, Ruobo Chu, Rencheng Zhang, Jinchao Xiao, Ran Tu

**Affiliations:** 1 Key Laboratory of Process Monitoring and System Optimization for Mechanical and Electrical Equipment (Huaqiao University), Fujian Province University, Xiamen 361021, China; yangkai1@hqu.edu.cn (K.Y.); cruobo@stu.hqu.edu.cn (R.C.); turan@hqu.edu.cn (R.T.); 2 Shenyang Institute of Automation, Chinese Academy of Sciences, Guangzhou 511458, China; xiaojiancao@sia.cn

**Keywords:** series arc fault, convolutional neural network, temporal domain visualization, gray image

## Abstract

AC arc faults are one of the most important causes of residential electrical wiring fires, which may produce extremely high temperatures and easily ignite surrounding combustible materials. The global interest in machine learning-based methods for arc fault diagnosis applications is increasing due to continuous challenges in efficiency and accuracy. In this paper, a temporal domain visualization convolutional neural network (TDV-CNN) methodology is proposed. The current transformer and high-speed data acquisition system are used to collect the current of a series of arc faults, then the signal is filtered by a digital filter and converted into a gray image in time sequence before being fed into TDV-CNN. Five different electric loads were selected for experimental validation with various signal characteristics, including vacuum cleaner, fluorescent lamp, dimmer, heater, and desktop computer. The experimental results confirm that the classification accuracy of the five loads’ work states in the ten categories could reach 98.7% or even higher by adjusting parameters perfectly. The methodology is believed to be reliable for series arc detection with relatively high accuracy and also has important potential applications in other fault diagnosis fields.

## 1. Introduction

Electrical fires could be induced by multiple reasons, e.g., arc fault, over current, leakage or overheating of electrical appliances. Arc faults often occur in residential power wires due to cable aging, loose electrical connections or virtual contacts, which are able to produce high temperatures exceeding 20,000 K [1] as an important factor for fire ignition. If effective arc fault identification measures are not taken in time to implement interruption, it may lead to a risk of electrical fire or even explosion [2]. According to the Fire Rescue Bureau of China Emergency Management Department on China fire and alarm situation in 2018 [3], electric fire ranks 1st among all kinds of fires, up to 34.6%, and more than 40% of electrical fires are caused by arc faults. More attention should be paid worldwide to protect against electrical fires caused by arc fault [4,5].

**Figure 1:**
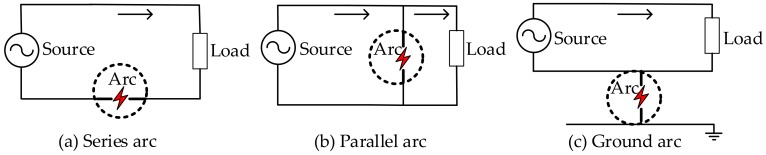
Types of arc faults.

According to the Under Laboratories (UL) Standard UL1699 [6], arcing is defined as a continuous luminous discharge of electricity across an insulating medium, usually accompanied by the partial volatilization of the electrodes, and is a very complicated electromagnetic reaction process [2]. Arc faults are categorized into three types as shown in Figure 1: series arc fault, parallel arc fault, and ground arc fault, among which the series arc occurs the most frequently [7,8,9].

The detection of the series arc needs to be designed according to the characteristics of the arc itself. Whereas the truth is that traditional electrical protection devices, such as overcurrent interrupters or ground fault interrupters, cannot trigger the protection due to the existence of arc impedance [4,10]. In addition, since arc faults are usually latent, intermittent, and transient, the state of the circuit should be monitored in real-time for early detection to prevent arc damage. An arc fault detection system is usually installed at the upstream of the power wire to prevent fires caused by arc faults.

In recent decades, the issues and related challenges of arc fault detection haves been extensively studied [3,4,5,11,12,13,14,15,16,17,18]. Previous studies have shown that arc faults exhibit some specific behaviors, such as arc radiation, sounds, light, temperatures, and voltages in circuits, etc. [11]. However, the positional uncertainty of arc faults limits the application of these characteristics. On the contrary, the convenience of the circuit current measurement makes it an ideal parameter for arc fault diagnosis.

As reported, many high-frequency components have been found in the currents of arc faults, with wide frequency bands [12,13,14]. Ji et al. [5] designed a bandpass filter with a frequency from 2.4 to 39 kHz to extract arc signals and verified that the db13 wavelet was the optimal mother wavelet to analyze the arc features using the discrete wavelet transform method. Zhao et al. [15] used stationary wavelet transform to remove the fundamental wave of the differential current signal, and used the maximum value of the high-level detail waveform as the arc detection basis. Artale et al. [16,17] proposed a high-resolution, low-spectrum analysis method for arc current harmonics, which enables good resolution and improves real-time recognition even in short observation windows. These methods have the advantage of conveniently extracting their features when decomposing the collected experimental signals. But, a limit was also found experimentally that these methods could not capture the individual characteristics of various loads, since the types of electrical appliances are changing and innovative.

The interest in machine learning-based methods for arc fault diagnosis applications is increasing due to their efficiency and accuracy. Some recent studies have achieved satisfactory results for AC series arc fault detection using machine learning-based methods. Gao et al. [18] presented a least squares support vector machine (SVM) and wavelet entropy method to realize arc fault recognition, which is using wavelet transform to extract the high-frequency signal generated near the zero-crossing arc fault current. Wang et al. [4] proposed a method for preprocessing the data using a sparse matrix and dictionary learning algorithm, and then performing training through a fully connected neural network (FCNN) layer. Although these methods can identify new load types, they cannot be ensured as the structure of the classifier applied is not designed according to the source data characteristics. Further, the characteristics of the acquired signals or the influence of preprocessing on the classifier algorithm showed a lack of consideration.

In this work, to prevent arc-caused fires in advance, a preprocessed method, converting load characteristics to a gray image for visualization is designed based on temporal domain visualization. Furthermore, a novel methodology, named TDV-CNN, is proposed to improve the accuracy and reliability of the series arc detector.

## 2. CNN Theory and Characteristics of Series Arc

### 2.1. Preliminary Theory of CNN

The TDV-CNN method proposed here is based on a convolutional neural network (CNN), which is similar to the traditional neural network in general, but more suitable for large-scale data processing. It is known that CNNs are designed to process data that come in the form of multiple arrays by moving certain filters to perform convolution operations with a specific stride.

The traditional neural network is composed of three parts: the input layer, hidden layer, and output layer. Each neuron only receives the input from the previous layer and outputs it to the next layer. This type of network can be thought of as multiple complexes of simple nonlinear functions from input space to output space [19,20].

For a 2D arrays  IN with one channel, the k filters with a size of $R_{f}\times C_{f}$, a step size s = 1, and the output feature map with a size of $H_{i}\times W_{i}$ and the convolutional operation for each filter can be calculated as follows:

(1) $$OUT_{k}\left(h_{i}\;,w_{i}\right)=\sum_{i\;=\;0}^{R_{f}-1}\;\sum_{j\;=\;0}^{C_{f}-1}IN\left(s\times h_{i}+i\;,\;s\times w_{i}+j\right)\times W_{k}\left(i,j\right)+B_{k},$$

where W  and B  are the weight coefficient matrix and the bias coefficient, respectively. $h_{i}$ and $w_{i}\;$ represent the row and column index of the output 2D array. Max pooling layer only keeps the maximum value within the specific square areas of the max-pooling kernel size, which is often employed after the CNN layer to reduce the output dimension. Therefore, the key information of the extracted features by the CNN layer can be preserved with reduced computation [21,22].

A leaky rectified linear unit (Leaky ReLu), as shown in Equation (2), is used as the activation function to alleviate the issue of vanishing gradient [22].

(2) $$f_{Leaky\;Relu}\left(x\right)\;=\;\;\left\{\begin{array}{c} 0.01\cdot x\;,\;\;\;x\leq 0,\\ \;\;\;\;\;x\;\;\;\;\;\;\;\;,\;\;\;x>0, \end{array}\right.$$

Usually, CNN’s optimization procedure is minimizing the cross entropy as close to 0 as possible by training with an amount of data. For a multiple classification case, the categorical cross entropy loss function based on a batch of data with size N, named  LOSS, can be defined as:

(3) $$LOSS=-\frac{1}{N}\overset{N}{\underset{i\;=\;1}{\sum }}\;\overset{K}{\underset{k\;=\;1}{\sum }}y_{ik}lg\frac{e^{\;x_{ik}}}{\sum_{k\;=\;1}^{K}e^{x_{ik}}}$$

where $y_{ik}$ is the truth category label while the $x_{ik}$ is the predicted output of the last layer. The dropout algorithm [12] is usually used to improve the generalization performance of the network.

### 2.2. Characteristics of Series Arc

**Figure 2:**
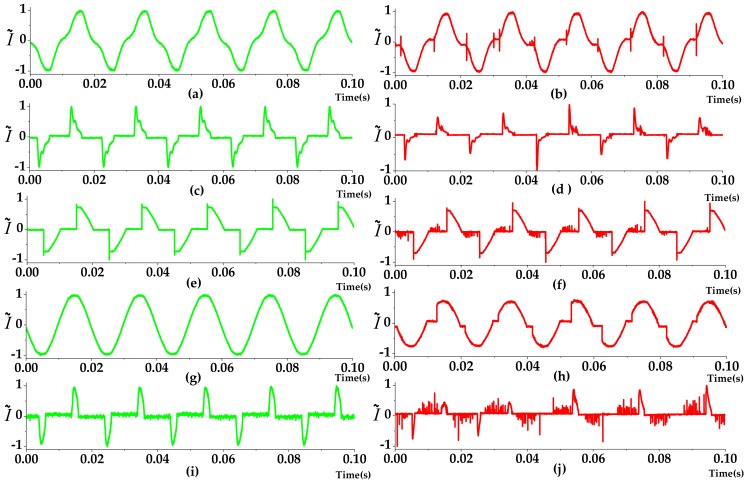
Current waveforms of various loads in normal state and arc state. (**a**) Vacuum cleaner_Normal; (**b**) Vacuum cleaner_Arc; (**c**) Fluorescent lamp_Normal; (**d**) Fluorescent lamp_Arc; (**e**) Dimmer_Normal; (**f**) Dimmer_Arc; (**g**) Heater_Normal; (**h**) Heater_Arc; (**i**) Computer_Normal; (**j**) Computer_Arc.

Figure 2 shows the current signal waveforms for the two circuit states (normal and arcing) from different typical loads. The waveforms for the normal state are different due to the power type. For example, the current waveform of the resistive load is generally sinusoidal, while the dimmer load has very obvious switching stripes.

When a series arc occurred in the circuit, abnormal behaviors such as amplitude distortion, impulse, or spiking, and increment of the harmonic component might be observed [4]. Especially, rich high-frequency characteristic signals can be found at arc current passing zero-crossing. Particularly, some normal states of load, such as a dimmer load, could imitate the arc state of some loads, like heater, causing misjudgment.

Therefore, methods based on the analysis of the load’s current or voltage generally cannot adapt to the requirements of arc fault detection. Designing a highly accurate and reliable arc fault detection method is still a challenge for the variety and complexity of arcing.

**Figure 3:**
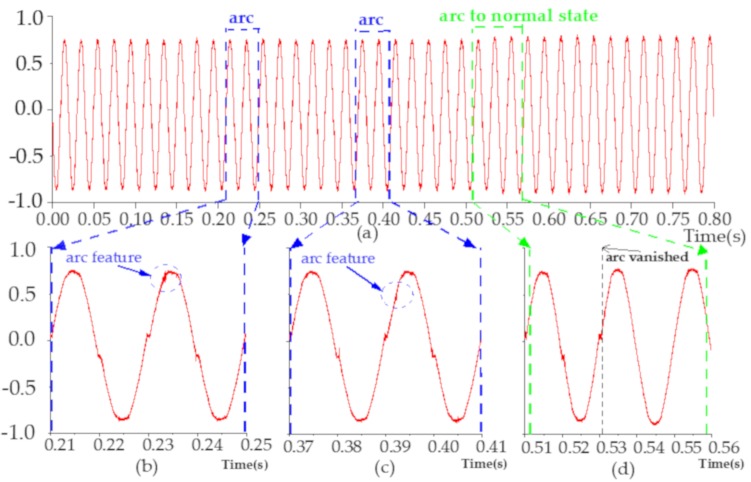
Heater load’s current. (**a**) From arc state to normal state; (**b**) Arc feature; (**c**) arc feature; (**d**) arc vanished randomly.

The heater load’s current waveform (after normalizing) collected at a sampling rate of 1 MS/s is shown in Figure 3a (with 10,000 data points in every half-cycle signal). As illustrated in Figure 3b,c, the arc high-frequency signals are generated in random positions. It should be noted that there are many redundant signals in massive data and their high-frequency features are often highly correlated. Moreover, if the signal’s high-frequency feature once appears in one place in the arcing zone, it could also appear anywhere. Hence, the features at different locations can share the same weights and detect with the same pattern in different parts of the data array, as the concept of discrete convolution [19,23]. For example, if an input image is scanned with a convolution kernel, the numerical value in the convolution kernel is called the weight. Each position of the image can be scanned by the same convolution kernel, and the weights of them are the same. Figure 3d shows that the generation of the arc is not continuous and has a certain randomness.

## 3. TDV-CNN Approach Development

The TDV-CNN method is proposed for series arc fault detection, which consists of a temporal domain visualization (TDV) layer, a convolutional neural network (ConvNet) layer, and a fully connected output (FCO) layer. These three layers work together to diagnose and distinguish the type of load from arc fault occurred in the circuit.

### 3.1. TDV Layer

**Figure 4:**
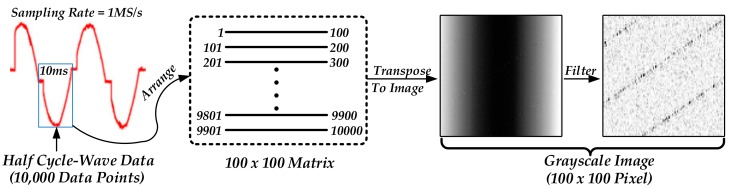
Preprocessing of experimental raw data.

The procedure of the TDV layer is illustrated in Figure 4, every half-cycle current signal that has 10,000 data points is regarded as a measurement object. Firstly, these data points will be preprocessed using Min-Max normalization [21] and further arranged into a matrix with a size of 100 × 100 according to the sequence of the temporal domain, as shown in Equation (4), where x is the normalized input, while $x_{raw}$ is the original collected raw data. Secondly, this matrix is transposed and converted to a gray image with a value range from 0 to 255 for visualization. Finally, the gray image is filtered by a 5–200 kHz Butterworth digital bandpass filter to effectively eliminate the fundamental frequency component, DC component, and noise generated by the AC mains.

(4) $$x\left(i,j\right)\;=\;\frac{x_{raw}\left[100\left(j-1\right)+i\right]-min\left(x_{raw}\right)}{\max\left(x_{raw}\right)-min\left(x_{raw}\right)}$$

**Figure 5:**
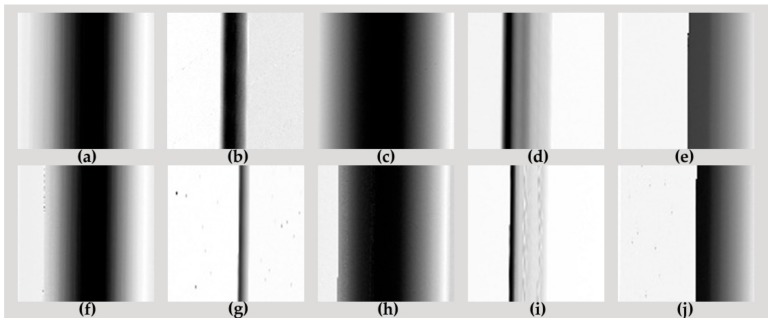
Normal and arc gray image before filtering. (**a**) Vacuum cleaner_Normal; (**b**) Computer_Normal; (**c**) Heater_Normal; (**d**) Fluorescent lamp_Normal; (**e**) Dimmer_Normal; (**f**) Vacuum cleaner_Arc; (**g**) Computer_Arc; (**h**) Heater_Arc; (**i**) Fluorescent lamp_Arc; (**j**) Dimmer_Arc.

**Figure 6:**
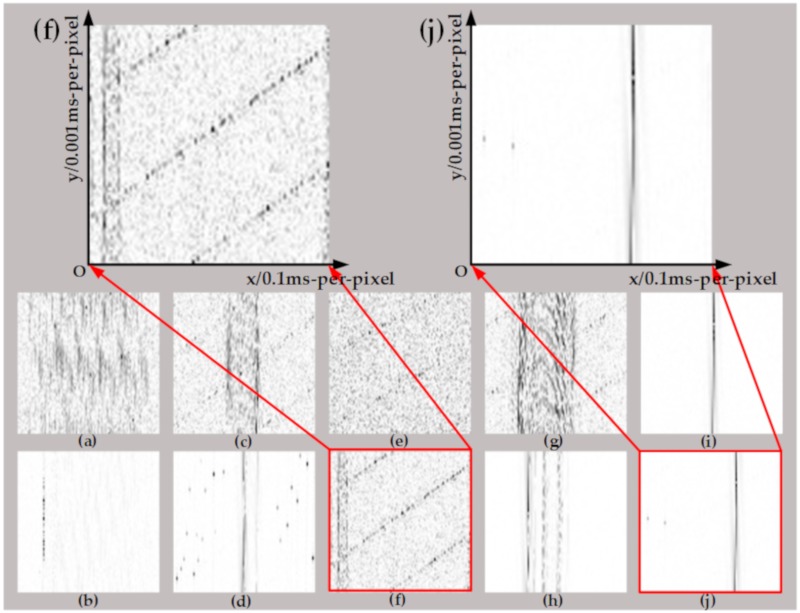
Arc and normal gray image after filtering. (**a**) Vacuum cleaner_Normal; (**b**) Computer_Normal; (**c**) Heater_Normal; (**d**) Fluorescent lamp_Normal; (**e**) Dimmer_Normal; (**f**) Vacuum cleaner_Arc; (**g**) Computer_Arc; (**h**) Heater_Arc; (**i**) Fluorescent lamp_Arc; (**j**) Dimmer_Arc.

After the half-cycle current waveform is preprocessed, both ten generated images and filtered images from five loads in different work states (normal or arc) are shown in Figure 5 and Figure 6, respectively.

As shown in Figure 5, the arc image and normal image both from the same load without filtering, are similar except a little noise. However, the difference between these images becomes quite clear after digital filter processing. To be specific, an obvious "flat shoulder" feature appears on the left and right sides of the heater image of the arc fault state (Figure 6f), which also showed obvious harmonic fringes and high-frequency noise after filtering. In addition, the filtered image of the dimmer (Figure 6j) has evident cut-off streaks because of the switching action of the silicon tube, leading to less high-frequency noise in the image of the arc state of the dimmer than the electric heater.

### 3.2. ConvNet Layer and FCO Layer

The ConvNet layer is composed of seven layers specifically designed to extract the loads’ work features. The preprocessed image from the TDV layer’s output is introduced as input and then the corresponding high-level features map is generated as output. The FCO layer consists of four fully connected layers with responsibility for reducing the dimension of the high-level features from the ConvNet layer’s output, and outputting the final predicted load work state.

**Figure 7:**
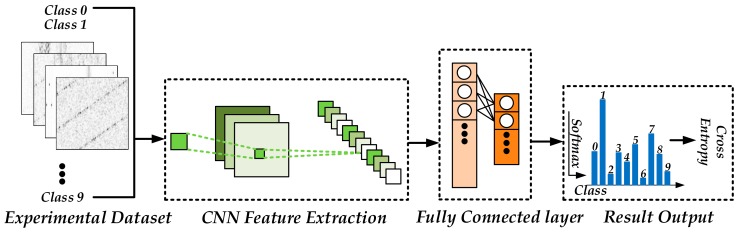
The flowchart of the proposed methodology (TDV-CNN).

The detailed parameters and architectures of TDV-CNN are summarized in Table 1. The flowchart of the TDV-CNN method is shown in Figure 7. After samples are converted to gray images with shrewd preprocessing, these feature images are fed into the ConvNet layer, which would learn high-level features from the input images automatically. Finally, the fully connected layer learns and produces the classification results as an output. Adam [24], an effective method for stochastic optimization, is used to optimize TDV-CNN in each iteration by calculating the cross entropy between the predicted output and the ground truth and aiming to optimize it.

## 4. Experimental Results and Analysis

### 4.1. Experimental Setup

**Figure 8:**
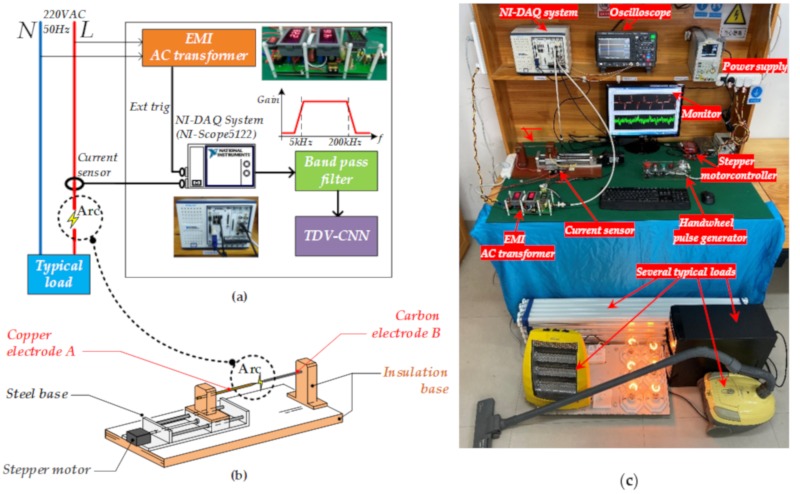
(**a**) Schematic of the experimental platform; (**b**) arc fault simulator; (**c**) actual experimental platform.

A series arc fault experimental platform was set up based on China’s standard GB14287.4-2014 [25] and American standard UL1699–2011 [6]. Figure 8a shows the schematic of the experimental platform. A data acquisition system (DAQ), which consists of a NI-PXIe-1071 chassis, a NI-PXIe-5122 module (witch 12 bit ADC resolution and 100 MS/s high sample rate), and a current transformer (CT, ZCT20-H, with cut-off frequency 250 kHz) is used to collect the loop current from the experimental circuit. The dual EMI power supply filter provides an external trigger signal from the electrical wiring to the DAQ to acquire half-cycle signal data entirely.

The adjustable arc generator with a carbon electrode and a copper electrode, as illustrated in Figure 8b, is designed and a 57 mm two-phase closed-loop stepper motor (YAKOTEC, YK257EC56E1, Shenzhen, China) with a programmable logic controller (PLC, Mitsubishi Electric, FX-3GA-40MT, Tokyo, Japan) is used to accurately control the distance between electrodes with a suitable speed. The complete experimental platform is given in Figure 8c. Typical load types and corresponding powers for this platform are shown in Table 2.

All raw data are digitized at a sampling frequency of 1 MS/s and each half-cycle signal is considered to be a complete identification object. A dataset with an overall size of 12,000 samples is used for training and testing, of which 6000 samples are arc fault states, while another 6000 samples are normal states. The training dataset and test set, obtained individually, have sample sizes of 10,000 and 2000, respectively.

The computer for data processing has four GPUs (NVIDIA GeForce GTX 1080Ti, Santa Clara, CA, USA) and dual CPUs (Intel Xeon E5-2678v3, Santa Clara, CA, USA). The model would be loaded after optimization training of TDV-CNN (~5 mins) and the response of TDV-CNN for real-time arc fault identification is relatively fast [4]. 

### 4.2. TDV-CNN’s Output with t-SNE Visualization Method

T-distribution stochastic neighbor embedding (t-SNE) [26] is used to analyze the output of TDV-CNN’s convolutional neural network layer, and the original 5408 dimensional space is projected onto the two-dimensional plane. The t-SNE is a machine learning method for dimensionality reduction. The superiority of t-SNE is that it preserves the data distances when they are mapped from the high-dimensional data to the low-dimension.

**Figure 9:**
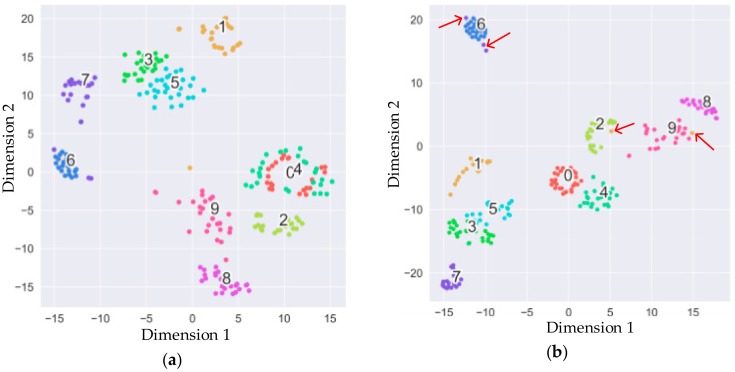
Visualization of the last layer with t-distribution stochastic neighbor embedding (t-SNE). (**a**) Initial stage of training; (**b**) late stage of training.

As shown in Figure 9, ten categories from five typical loads’ work states correspond to the projection of ten points groups with different colors in two-dimensional space. The colorful point’s label for each load is shown in Table 3. Label 1, 3, 5, 7, and 9 are arc states, and label 0, 2, 4, 6, and 8 are normal states, respectively.

In the initial stage of training, Figure 9a shows that most categories are clearly separated from each other, and only a small number of points are located in other categories for similar features. While, for the late stage, the various typical loads begin to shrink into a group and move away from each other, indicating that the ConvNet layer has an excellent extraction of arc image features. However, there are also some points that are assigned to the wrong location. For example, a few points of category 7 still appear around the point-group belonging to category 6, as shown in Figure 9b. Categories 6 and 7 belong to the dimmer load, and its arcing state has less high-frequency characteristic signals than others, which results in unsatisfied low-dimensional analysis.

### 4.3. Test Verification and Analysis

**Figure 10:**
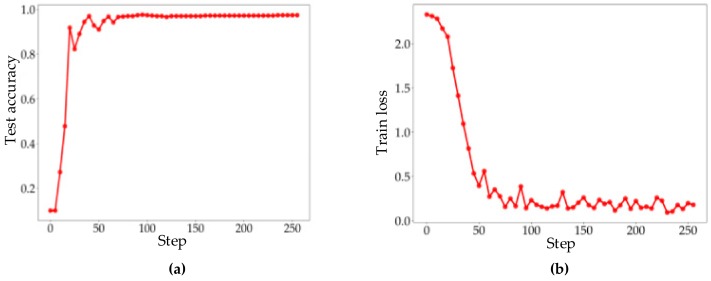
(**a**) Test accuracy; (**b**) train loss.

The train loss function and test accuracy trend of TDV-CNN are shown in Figure 10a,b respectively. In the initial stage of training, the loss function decreases rapidly with fast-increasing test accuracy. From the 60th iteration, the variation rate of test accuracy and train loss begin to decrease slowly. The loss function is still in the falling range and the test accuracy is slowly rising, indicating that the network continues to optimize. After 100 iterations, the network has tended to be stable.

**Figure 11:**
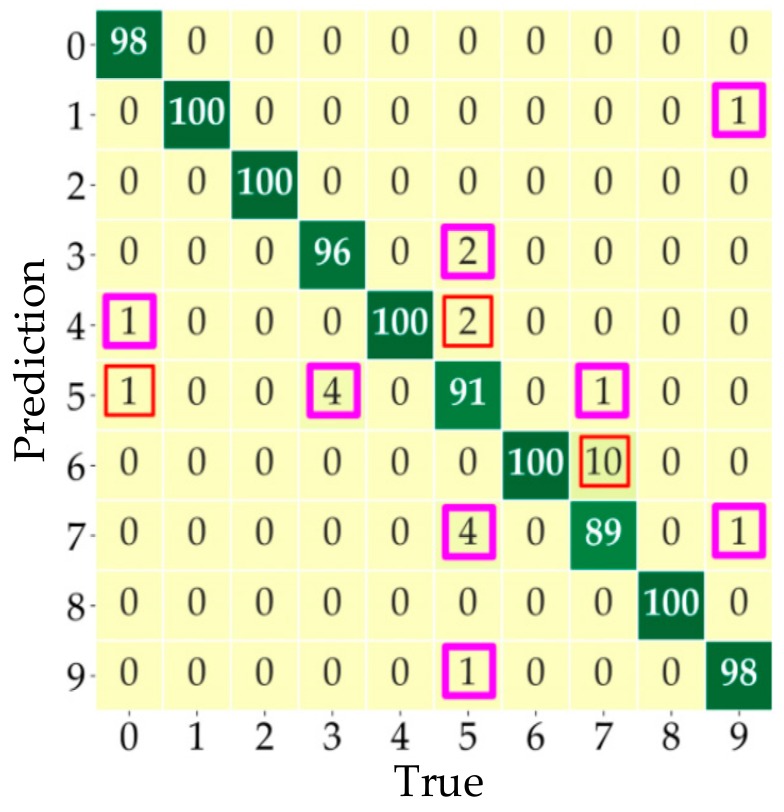
The confusion matrix for multi-loads.

The typical loads listed in Table 2 are employed to evaluate the capability of the TDV-CNN method as shown in Table 4. Figure 11 presents the general distribution of the classification confusion matrix of the test data set with detailed values, in which labels and corresponding loads can be found in Table 3. The general prediction accuracy of each sample is 97.2%. Among 10 categories from the five typical loads, the lowest accuracy is the arc state of the dimmer load with 89%. This result confirms that the TDV-CNN method has good recognition accuracy.

In Figure 11, it should be noted that the samples framed in pink could be accepted as correct predictions, if focusing on whether the arc fault occurs but ignoring the load type. The work state of the samples framed in pink is predicted correctly by TDV-CNN but the type is predicted wrong. Consequently, the prediction accuracy of arc detection is further increased to 98.7%, if ignoring the load type.

**Figure 12:**
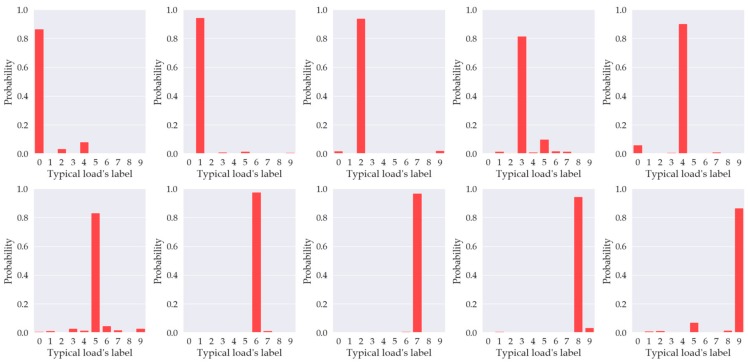
Distribution of probabilities of the different load types.

Figure 12 presents the distribution of probabilities of the different load types under the correct prediction, in which labels and corresponding loads can be found in Table 3. If the prediction of the TDV-CNN algorithm is correct, the probability distribution of the test load is more than 80%. However, if the load is combined, the situation will be different. Although the algorithm can detect whether there is arc fault in the circuit most of the time, the identification rate of arc faults will reduce a little. Improving the identification rate is a future research target.

The classification precision for normal states (category 0, 2, 4, 6, and 8) are very close to 100%. The suggested reason for this is that the features of the normal state are regular and steady. Hence, TDV-CNN could learn the loads’ inherent features in the train set and perform with satisfactory accuracy in the test set. On the other hand, the classification accuracy of arc states (category 1, 3, 5, 7, and 9) is lower than the normal state of each corresponding load type. Since TDV-CNN may not be able to fully capture all possible high-level features of loads’ arc states, patient adjustment of model parameters would be necessary to further improve TDV-CNN’s performance. The next step is to implement the TDV-CNN method in a field-programmable gate array (FPGA) to realize real-time arc fault detection.

### 4.4. Comparison with Prior Methods

In the comparison with some typical methods from recent literature, the detection accuracy, application range, and recognition of the exact arcing type are contrasted to evaluate the TDN-CNN method and show the properties, as summarized in Table 5.

In the aspect of detection accuracy, there is no one way to ensure perfect accuracy, and a certain false positive rate is acceptable. It is generally believed that an accuracy rate of more than 95% can be considered to meet the design requirements. In respect to recognizing the exact arcing type, it is not necessary to point out what the load type is, according to relevant standards, but this would help users to know the approximate location of the arc fault, which may be a future research interest.

Yang et al. [14] considered autoregressive bi-spectrum analysis to analyze common series arc fault features and used a least-squares support vector machine to accurately identify series arc faults from the load states, and their method works well to achieves an accuracy of up to 97%.

The method in [4] used a combination of neural network and sparse representation, which has high accuracy and can identify the type of load, but the complexity of this method increases. In contrast, the pre-processing method used by the TDV-CNN method is simple, and it is not necessary to combine other algorithms to extract the features of the data, so that the recognition time can be shorter, and the pre-processing method is easily implemented in the real-time operating system.

The method in [27] proposed that the detection accuracy of the arc fault based on the combination of FFT, CZT, and DB4 can reach 99.85%. Such high accuracy is derived from the analysis of the frequency distribution band for specific loads. But the fact is that there is no general frequency distribution band that recognizes arc faults from the circuit, since the abnormality may exist in and out of the frequency band. The TDV-CNN method is not affected by the various frequency distributions generated by the various loads and can be used to distinguish various load types of different operating states.

In particular, it is reasonable to take the signal on a half-cycle scale for incipient discontinuous and continuous arcing detection, which fulfills the corresponding requirements of counting the arcing half-cycles in 0.5 s by GB 14287.4–2014 standard [25] and UL1699 standard [6].

## 5. Conclusions

To identify the series arc faults effectively, firstly, a specific experimental platform is built to collect the current of normal and arc fault states. Secondly, the TDV-CNN methodology is proposed to extract various load types of different work states from each other. When a series arc fault occurs in the circuit, a large number of high-frequency signals would be generated randomly. The arc fault’s high-frequency signal features are the same concept as the object features in the image, e.g., redundancy, local correlation, and randomness, if the arc fault is transposed into a characteristic gray image. This concept is the theoretical basis for the TDV-CNN method.

The experimental results confirm the TDV-CNN methodology’s effectiveness and good accuracy for series arc fault detection. If the load type is ignored, the detection accuracy reaches 98.7% and could be higher by adjusting the parameters perfectly. This study will help with the development of arc fault detection and other fault diagnoses.

## Figures and Tables

**Table 1:** The architectures and parameters of TDV-CNN.

No.	Layer type	Activation	No. of Kernel	Dropout	Padding	Kernel size	Stride	Output
1	Conv.	Leaky ReLu	6	No	2	5 × 5	1	104 × 104 × 6
2	Maxpooling	-	-	No	-	2 × 2	2 × 2	52 × 52 × 6
3	Conv.	Leaky ReLu	12	No	2	5 × 5	1	52 × 52 × 12
4	Maxpooling	-	-	No	-	2 × 2	2 × 2	26 × 26 × 12
5	Conv.	Leaky ReLu	24	No	2	5 × 5	1	26 × 26 × 24
6	Maxpooling	-	-	No	-	2 × 2	2 × 2	13 × 13 × 24
7	Linear	Leaky ReLu	1	0.2	-	4056	-	4056
8	Linear	Leaky ReLu	1	0.2	-	512	-	512
9	Linear	Leaky ReLu	1	0.2	-	64	-	64
10	Linear	Softmax	1	No	-	10	-	10

Conv.: two-dimensional convolutional layer.

**Table 2:** Typical loads.

Number	Load Name	Power (W)	Load Type
1	Vacuum cleaner	1200	Inductive
2	Fluorescent lamp	480	Capacitive
3	Dimming lamp	1000	Others
4	Heater	800	Resistive
5	Desktop computer	350	Switching

**Table 3:** Typical loads and the corresponding labels.

Load Name	Load Type	Work State	Data Set Label
Heater	Resistive	Normal	0
		Arc	1
Vacuum cleaner	Inductive	Normal	2
		Arc	3
Desktop Computer	Switching	Normal	4
		Arc	5
Dimmer	Others	Normal	6
		Arc	7
Fluorescent lamp	Capacitive	Normal	8
		Arc	9

**Table 4:** Classification results of TDV-CNN method.

Category	0	1	2	3	4	5	6	7	8	9
Identification accuracy	98%	100%	100%	96%	100%	91%	100%	89%	100%	98%
Prediction accuracy of each sample: 97.2%										
Prediction accuracy (ignore the load type): 98.7%										

**Table 5:** Properties comparison with prior methods.

Methods	Detection Accuracy	Recognize the Exact Arcing Type
Yang et al. [14]	97.0%	×
Wang et al. [4]	94.3%	√
Hien et al. [27]	99.85%	×
TDV-CNN method	98.7%	√
